# Microscopic Transsphenoidal Resection of Giant Pituitary Adenomas: Analysis of the Factors Limiting the Degree of Resection in 73 Cases

**DOI:** 10.3389/fneur.2022.880732

**Published:** 2022-05-27

**Authors:** Zhijie Pei, Jiaxing Wang, Shuwen Mu, Tianshun Feng, Meina Wang, Shentong Yu, Liangfeng Wei, Yi Fang, Shousen Wang

**Affiliations:** ^1^Department of Neurosurgery, Fuzong Clinical Medical College of Fujian Medical University, Fuzhou, China; ^2^Department of Neurosurgery, The First Hospital of Yichang, The People's Hospital of China Three Gorges University, Yichang, China; ^3^Department of Neurosurgery, Oriental Hospital Affiliated to Xiamen University, Fuzhou, China; ^4^Department of Pathology, The 900th Hospital, Fuzong Clinical Medical College of Fujian Medical University, Fuzhou, China; ^5^Department of Neurosurgery, The 900th Hospital, Fuzong Clinical Medical College of Fujian Medical University, Fuzhou, China

**Keywords:** giant pituitary adenoma, microscopic transsphenoidal surgery, cavernous sinus invasion, pituitary surgery, gross-total resection

## Abstract

**Purpose:**

To analyze the risk factors affecting the gross-total resection of giant pituitary adenomas using a transsphenoidal approach under a microscope to provide a reference basis for formulating an appropriate surgical strategy.

**Methods:**

The clinical data of patients who underwent microscopic transsphenoidal resection of giant pituitary adenomas in a single center from January 2011 to December 2020 were retrospectively analyzed. Based on magnetic resonance imaging and surgical records, the predictive factors affecting the gross-total resection of giant pituitary adenomas under microscopy were determined through univariate and multivariate analyses.

**Results:**

A total of 73 patients with giant pituitary adenomas underwent transsphenoidal microsurgery. Gross-total resection was performed in 19 cases (26%), subtotal resection in 31 cases (42%), partial resection in 21 cases (29%), and the degree of resection was <50% in only two cases (3%). After binary logistic analysis, it was found that it was more difficult to completely remove giant pituitary adenomas with a Knosp grade 3–4 [odds ratio (OR) = 0.214, 95% confidence interval (CI): 0.05–0.917; *P* = 0.038], greater proportion of tumor suprasellar volume (odds ratio = 0.937, 95% confidence interval: 0.898–0.978; *P* = 0.003), and intraoperative evidence of invasion of the cavernous sinus (odds ratio = 0.187, 95% CI: 0.039–0.898; *P* = 0.036).

**Conclusion:**

It is difficult to remove a giant pituitary adenoma invading the cavernous sinus completely with a higher degree of invasion of the suprasellar region using microscopic transsphenoidal surgery. The combined application of multiple surgical methods can help to improve the degree of resection during a single operation.

## Introduction

Giant pituitary adenomas (GPA) are pituitary adenomas with a diameter >40 mm, which account for 5–14% of all pituitary adenomas, and have a radical resection rate of <50% (1). Due to the huge size and invasive nature of these tumors, the postoperative mortality and disability rates for the transsphenoidal approach and craniotomy for GPA resection are 1.5–18.7% and 10.4–23.2%, respectively (2, 3). Approximately 54.3–91.7% of GPA are clinically non-functional adenomas at the time of diagnosis, and damages to the vision, visual field, and hydrocephalus due to the space-occupying effect are the most common and serious complications (2). Apart from drug treatment for prolactin cell adenomas, surgery is still the preferred treatment. Although microscopic or endoscopic transsphenoidal surgery (TSS) is a widely used treatment option, safe gross-total resection (GTR) of GPA remains a huge surgical challenge (4). Therefore, the main purpose of surgery is to remove the tumor to the greatest degree while ensuring safety and relieving the space-occupying effect as much as possible. The residual tumor can be controlled through adjuvant therapy (5). However, improving the degree of resection of GPA can result in more satisfactory treatment results and prolong the recurrence time. Analyzing the risk factors that limit the degree of GPA resection under the microscope during transnasal TSS can help to fully evaluate the tumor's characteristics and improve the operation plan before surgery, thus ensuring the safe and satisfactory resection of the tumor.

## Materials and Methods

### Study Design and Patient Characteristics

This study retrospectively analyzed the clinical data of patients with GPA who were treated with microscopic TSS in the neurosurgery department between January 2011 and December 2020. Data was collected and analyzed over 1 year. The recorded clinical data included age, sex, magnetic resonance imaging (MRI) findings before and 3 months after surgery, surgical records, tumor pathological type, and hormone level. All patients or their families gave written informed consent for the clinical procedures and inclusion in the study. All procedures included in the study were approved by the Ethics Committee at Fujian Medical University, China.

Inclusion criteria are: (1) tumor diameter >40 mm; (2) the microscopic transnasal transsphenoidal approach was used in the primary surgery; (3) the operation was performed by the same surgeon; (4) pituitary adenoma was confirmed by pathology after the operation; (5) complete preoperative and postoperative imaging data and detailed surgical records were available.

The exclusion criteria were as follows: (1) secondary operation or craniotomy, (2) preoperative radiotherapy treatment, and (3) pathologically confirmed pituitary adenoma with other lesions in the sellar region.

### Image Evaluation

**Figure 1:**
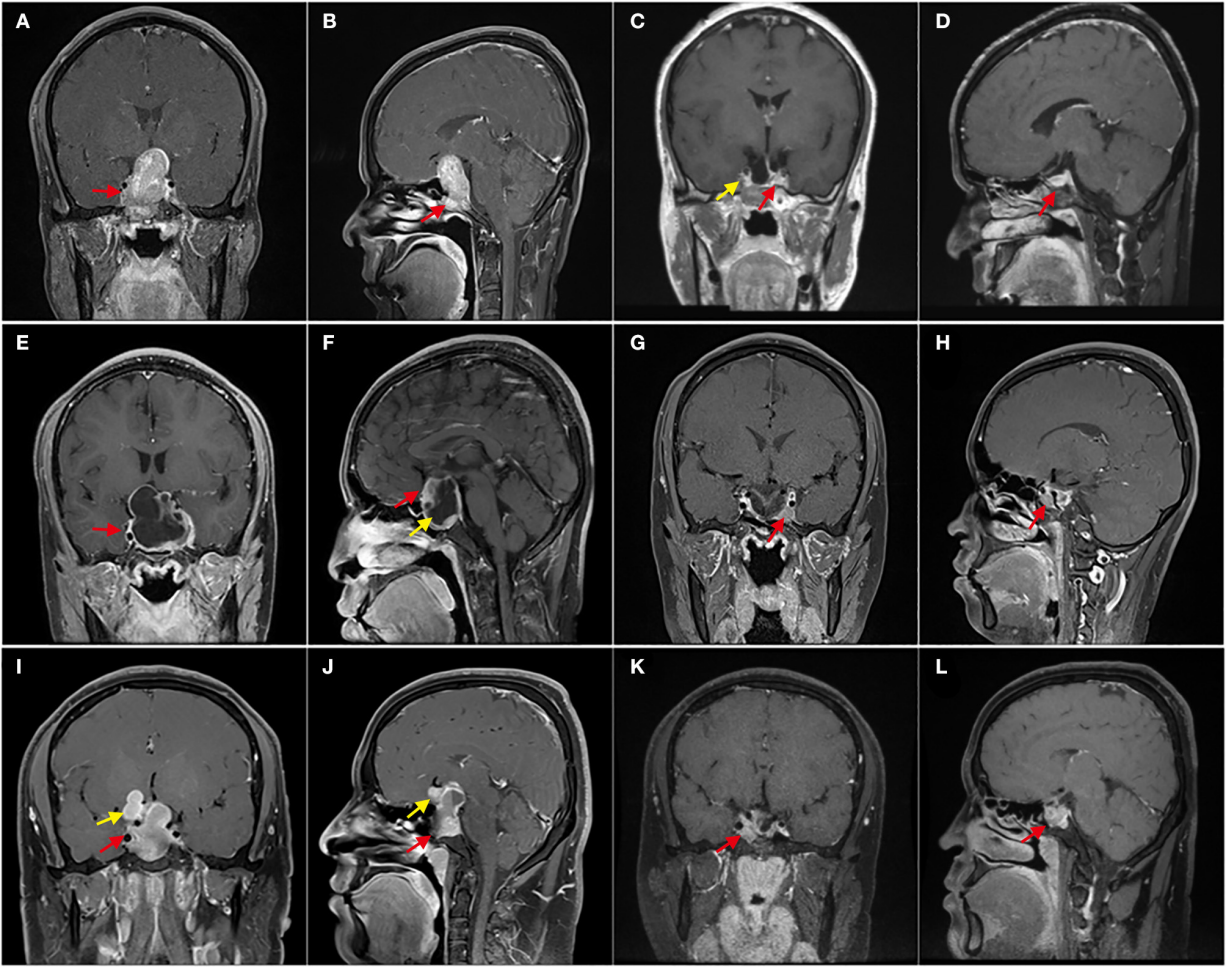
Enhanced T1 MRI with different resection degrees. **(A–D)** Patient 1, Preoperative MRI showed that the tumor invaded the sphenoid sinus downward and severely squeezed the right cavernous sinus (red arrow); 3 months after the operation, MRI showed that there was no residue in the right cavernous sinus (yellow arrow), and the normal pituitary was located on the left side of the sellar area (red arrow); It is gross-total resection. **(E–H)** Patient 2, E shows GPA with giant cystic, and F shows solid components (red arrow) and cystic components (yellow arrow); **(G,H)**, 3 months after the operation, MRI shows a few residual tumors in the left cavernous sinus (red arrow). With a residual of about 5%, which was subtotal resection. **(I–L)** Patient 3, the tumor was lobulated, extended to the anterior and suprasellar (yellow arrow), and invaded the sphenoid sinus (red arrow); **(K,L)** shows that 3 months after the operation, the residual tumor was mainly located in the left cavernous sinus (red arrow), and the residual was about 25%, which was partially resectioned.

Using the German Siemens 3.0T Magnetom trio Tim magnetic resonance scanner, all patients underwent pituitary MRI before and 3 months after the operation. According to the sagittal and axial position of T1WI, coronal and axial position of T2WI, coronal position and enhancement sequence of flair, we evaluated the tumor diameter, suprasellar extension height and grade, suprasellar tumor volume proportion, Knosp grade, tumor shape or lobulation, invasion of the middle cranial fossa, preoperative hydrocephalus, and the resection degree. The degree of tumor resection was divided into GTR, subtotal resection (residual tumor volume <20%), and partial resection (residual tumor volume <50%) (6). Figure 1 shows the definition of various resection degrees. The improved ellipsoid volume calculation formula ([a ^*^ b ^*^ c] / 2) was used to calculate the residual tumor volume and suprasellar tumor volume; a, b, and c represent the maximum diameters of the three dimensions, respectively. The extension degree of GPA on the sella, before the sella, and behind the sella was graded using the SIPAP classification of Edal et al. (7). The classification for suprasellar extension is as follows: Grade 0, the tumor did not reach the suprasellar cistern; Grade 1, the tumor reached the suprasellar cistern and did not reach the optic chiasm; Grade 2, the tumor reached the optic chiasm exerting pressure on it; Grade 3, apparent cross compression deformation; Grade 4, at least one lateral interventricular foramen is squeezed and leads to obstructive hydrocephalus. For anterior and posterior sellar extension, the classification is Grade 0, the anterior boundary of the tumor did not exceed the vertical line of the sellar tubercle, and the posterior boundary did not reach the back of the sella; Grade 1, the anterior boundary exceeds the vertical line of the sellar tubercle, and the posterior boundary reaches the back of the sellar dorsum. The cavernous sinus invasion (CSI) was graded by the Knosp grade (8), and patients with Knosp grade 0–2 were regarded as the low-level group, while those with Knosp 3–4 were considered the high-level group. The tumor morphology was divided into two types: lobulated and undifferentiated. According to the compression of the third ventricle and expansion of the lateral ventricle, GPAs were divided preoperatively into the hydrocephalus and non-hydrocephalus groups. If the GPA expands laterally, or if the tumor extends into the middle cranial fossa, it is regarded as the invasion of the middle cranial fossa. If not, it is considered not invasive. All measurement data and information were collected independently by two individual researchers, and then the average value was taken. Any differences were resolved through discussion and consensus.

### Surgical Methods and Intraoperative Records

The patient's head was kept about 5 cm higher than the feet on the operating table. The head was tilted back so that the upper incisors were in the same vertical line as the external auditory meatus. After iodophor disinfection of the bilateral nasal cavities, norepinephrine saline cotton tablets were used to fill the cavities for 3 min. The swelling fluid was injected into the nasal septum mucosa, followed by peeling the unilateral nasal septum mucosa up to the opening of the sphenoid sinus. Next, the anterior wall of the sphenoid sinus was exposed after the bone of the nasal septum was expanded by the spreader. A high-speed grinding drill was used to successively grind the bone of the anterior wall of the sphenoid sinus and sellar base, followed by crossing the dura mater at the sellar base, scraping the tumor tissue from the sellar base with a scraping ring and tumor tweezers, and sucking up the small pieces of tissue with a suction device. It is necessary to reduce tearing and prevent early damage to the sellar diaphragm. After the tumor in the upper part of the sella slowly sinks into the sellar area, it is removed step-by-step. Bleeding in the operation area should be stopped by compression with a cotton or gelatin sponge, and bipolar electrocoagulation should be avoided as much as possible. After tumor resection, blunt instruments such as the curettes were used again to explore the integrity of the medial wall of the bilateral cavernous sinus.

If the inner wall of the cavernous sinus is damaged, the tumor inside the cavernous sinus can be moderately scraped out through a breach with curettes. In cases of cerebrospinal fluid (CSF) leakage caused by sellar diaphragm damage during surgery, an artificial dura mater was used to repair the sellar diaphragm. The tumor cavity was filled with sufficient gelatin sponge to moderately compress the suprasellar diaphragm and artificial dura mater. After hemostasis, the sellar floor was repaired using artificial materials and biological protein glue, and the surgical channel was closed after leaving a rubber drainage tube in the sphenoid sinus. The nasal mucosa was reset, the nasal cavity was filled with an expanded sponge, and the expanded sponge was pulled out on the 3rd day after the operation.

The operator evaluated the texture, blood supply, and adhesion of the tumor boundary during the operation and recorded whether any CSF leakage occurred. CSF leakage is considered evidence of damage to the sellar diaphragm during surgery. All the above factors were grouped using binary classification: texture was divided into “soft” and “tough,” and tumor blood supply was classified into “poor” and “good.” Boundary adhesion and intraoperative CSF leakage were divided into “yes” and “no.” All procedures were performed by one surgeon and the chief surgeon (SS. W), including complete TSS resection of more than 1,000 pituitary adenomas. The surgeon personally judged the clinical characteristics of the tumor and completed the surgical records.

### Statistical Methods

SPSS 22.0 software (SPSS Inc., Chicago, Illinois, USA) was used for statistical analysis. Clinical data were expressed as means ± standard deviation, medians (interquartile range), frequencies, and percentages. The measurement data conforming to a normal distribution were compared using the independent sample *t*-test, and counting data were compared using the χ^2^ test or Fisher exact test, while the data with a non-normal distribution were compared using the Wilcoxon rank sum test. Binary logistic regression analysis was used to determine the independent risk factors limiting the GTR of GPA and expressed as odds ratio (OR) and confidence interval (CI). Differences were considered significant when the two-tailed *P*-value was <0.05.

## Results

### General Results

The basic information, clinical features, and tumor resection results of the GPA patients are shown in Table 1. There were 73 patients, including 46 men and 27 women, with an average age of 49.05 ± 12.48 years. According to the preoperative MRI, the mean diameter of the tumor was 47.11 ± 7.09 mm, 21 patients had a low Knosp grade (grade 0–2), and 52 patients had a high Knosp grade (grade 3–4). The medial wall of the cavernous sinus was intact during surgery in 36 patients; that is, these patients had no invasion of the cavernous sinus, while 37 had invasion of the cavernous sinus. The most common pathological subtype was gonadotroph adenoma (44 cases), with only one case of thyrotroph adenomas and null cell adenoma each. Thirty patients had hypertension, 15 had diabetes, and 32 had hyperlipidemia. The results are presented in Table 1.

**Table 1:** Demographic characteristics of 73 patients who underwent microscopic transsphenoidal surgery for giant pituitary adenomas.

**Variable**	**No**.
Age (years)	**49.05** **±12.48**
Sex	
Male	**46 (63%)**
Female	**27 (37%)**
Tumor size (mm)	**47.11** **±7.09**
Suprasellar tumor height (mm)	**19.65** **±7.92**
Proportion of suprasellar tumor volume (%)	**44.38** **±15.54**
Tumor texture	
Soft	**60 (82.2%)**
Tough	**13 (17.8%)**
Tumor blood supply	
Poor	**24 (32.9%)**
Good	**49 (67.1%)**
Tumor boundary adhesion	
No	**36 (49.3%)**
Yes	**37 (50.7%)**
Intraoperative confirmed CSI	
No	**36 (49.3%)**
Yes	**37 (50.7%)**
Preoperative hydrocephalus	
No	**58 (79.5)**
yes	**13 (20.5)**
Intraoperative cerebrospinal fluid leakage	
No	**57 (78.1%)**
Yes	**16 (21.9%)**
Tumor lobulation	
No	**44 (60.3%)**
Yes	**29 (39.7%)**
Invasion of middle cranial fossa	
No	**52 (71.2%)**
Yes	**21 (28.8%)**
Suprasellar extension grade (SIPAP)	
0–2 grade	**28 (38.4%)**
3–4 grade	**45 (61.6%)**
Anterior and posterior sellar extension grade (SIPAP)	
0 grade	**51 (69.9%)**
1 grade	**22 (30.1%)**
Knosp grade	
0–2 grade	**21 (28.8%)**
3–4 grade	**52 (71.2%)**
Pathological type	
Gonadotroph adenoma	**44 (60.3%)**
Lactotroph adenomas	**10 (13.7%)**
Somatotroph adenomas	**5 (6.8%)**
Corticotroph adenomas	**5 (6.8%)**
Thyrotroph adenomas	**1 (1.4%)**
Null cell adenoma	**1 (1.4%)**
Plurihormonal adenomas	**7 (9.6%)**

*CSI, cavernous sinus invasion; SIPAP, suprasellar, infrasellar, parasellar, anterior, and posterior; Values are expressed as mean ± standard deviation, median (interquartile range), or number (%). Significant P-values are shown in bold*.

### Clinical Factors Affecting the Degree of GPA Resection

Of the total 73 patients, 19 (26%) underwent GTR of GPA, 31 (42%) underwent subtotal resection (residual tumor volume <20%), 21 (29%) had partial resection (residual tumor volume <50%), and in only two (3%) patients, the degree of resection was <50% Intraoperative evaluation of CSI, the proportion of the tumor suprasellar volume, Knosp grade, and middle cranial fossa invasion were the risk factors affecting the degree of GPA resection. A larger volume of the suprasellar part of the tumor and a Knosp grade of 3–4 decreased the likelihood of completely removing the tumor. Preoperative MRI showed whether the tumor broke through the cavernous sinus and grew into the middle cranial fossa. The tumor was less likely to be removed if the CSI was confirmed during the operation. Sex, age, tumor texture, blood supply, boundary adhesion, SIPAP grade (suprasellar, presellar, and postsellar extension), diameter, lobulation, preoperative hydrocephalus, and intraoperative CSF leakage were not related to the degree of GPA resection. At the same time, there was no difference in the degree of resection between GPAs with a secretory function and those without a secretory function (*P* > 0.05). The results are presented in Table 2.

**Table 2:** Univariate factor analysis of risk factors affecting the degree of GPA resection.

**Factors**		**Gross-total resection (*n* = 19)**	**No total resection (*n* = 54)**	***t*/χ^2^/*z***	***P-*value**
Age		48.7 ± 15.5	49.2 ± 11.4	−0.13	0.898
Suprasellar tumor height (mm)		19.9 ± 5.7	19.6 ± 8.6	0.21	0.837
Proportion of suprasellar tumor volume (%)		37.9 ± 12.2	46.7 ± 17.3	−2.04	0.046^*^
Longest diameter of the tumor		43.12 (40.87, 45.68)	46.76 (42.07, 50.96)	−1.82	0.068
Sex	Male	14	32	1.26	0.26
	Female	5	22		
Tumor texture	Soft	15	45	0.18	0.731
	Tough	4	9		
Tumor blood supply	Poor	6	18	0.02	0.889
	Good	13	36		
Tumor boundary adhesion	No	11	25	0.756	0.384
	Yes	8	29		
Intraoperative confirmed CSI	No	15	21	9.02	0.003^*^
	Yes	4	33		
Knosp grade	0–2	11	10	10.63	0.001^*^
	3–4	8	44		
Suprasellar extension grade (SIPAP)	0–2	5	23	1.58	0.21
	3–4	14	31		
Preoperative hydrocephalus	No	18	40	2.52	0.112
	Yes	1	4		
Intraoperative cerebrospinal fluid leakage	No	17	40	1.15	0.210
	Yes	2	14		
Anterior and posterior sellar extension grade (SIPAP)	0	16	35	2.51	0.113
	1	3	19		
Tumor lobulation	Yes	4	25	3.74	0.53
	No	15	29		
Invasion of the middle cranial fossa	No	18	24	6.92	0.009^*^
	Yes	1	20		
Clinical diagnosis	FPA	1	12	1.73	0.19
	NFPA	18	42		

*CSI, cavernous sinus invasion; GPA, giant pituitary tumors; FPA, functioning pituitary adenoma; NFPA, non-functioning pituitary adenoma; SIPAP, suprasellar, infrasellar, parasellar, anterior, and posterior; Values are mean±standard deviation, median (interquartile range), or number (%). Significant P-values are shown in bold*.

* *P < 0.05*.

### Relationship Between the Pathological Subtypes of GPA and Degree of Resection

According to the 2017 World Health Organization pathological classification of pituitary adenomas, all pathological subtypes were observed among the 73 cases. Table 1 shows the specific distribution. The chi-square test results showed no significant difference in the degree of resection among the different pathological subtypes of GPA (*P* > 0.05). The results are presented in Table 3.

**Table 3:** Analysis of the relationship between the pathological subtypes and degree of resection of GPA.

**Pathological tumor type**	**Gross-total resection (*n* = 19)**	**No total resection (*n* = 54)**	**χ^2^**	***P*-value**
Null cell adenoma	0	1	5.26	0.54
Corticotroph adenomas	3	2		
Somatotroph adenomas	0	5		
Lactotroph adenomas	2	8		
Thyrotroph adenomas	0	1		
Gonadotroph adenoma	12	32		
Plurihormonal adenoma	2	5		

### Independent Risk Factors

**Figure 2:**
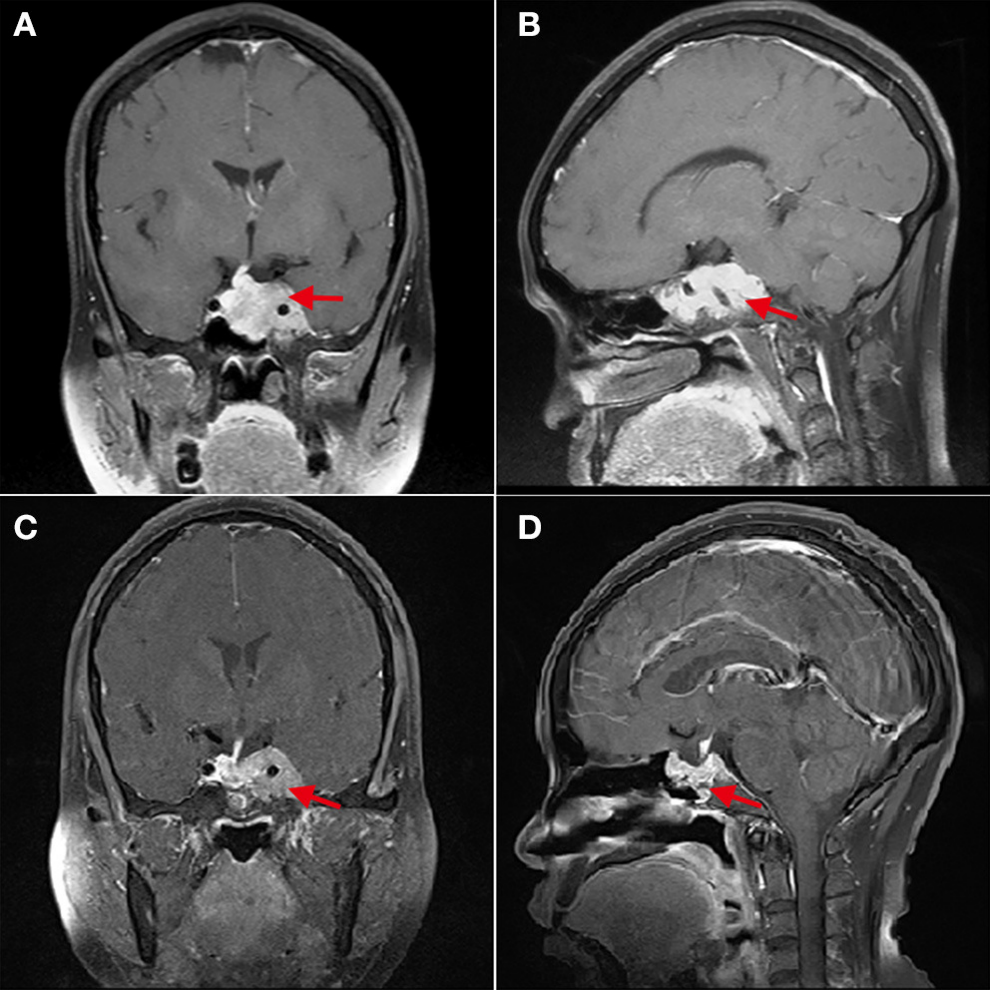
The cavernous sinus invasion limits the gross-total resection of GPA. **(A,B)** Patient 4, **(A)** shows that the left cavernous sinus is filled with tumor, and the internal carotid artery is surrounded (red arrow); **(B)** shows that the sphenoid sinus is also filled with tumor tissue. **(C,D)** Three months after operation, **(C)** shows the residual tumor in the left cavernous sinus (red arrow).

**Figure 3:**
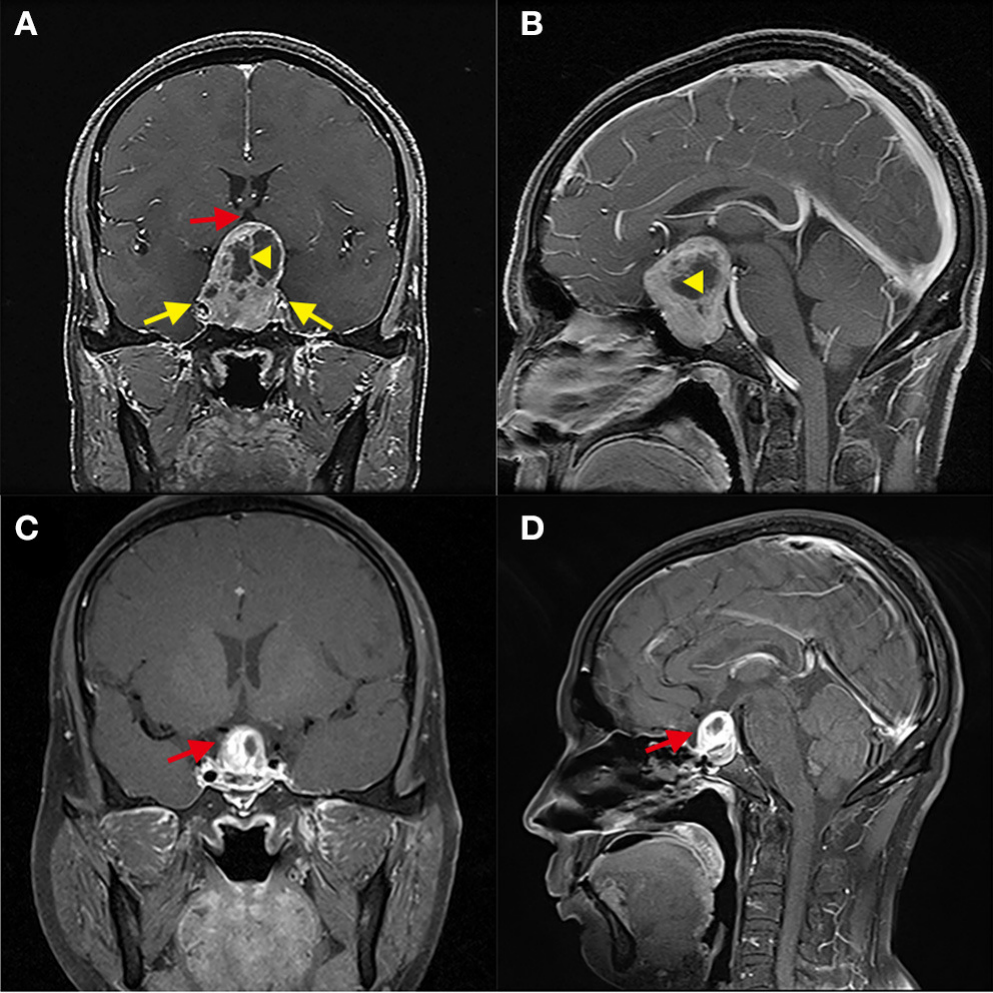
Greater proportion of tumor suprasellar volume limits the gross-total resection of GPA. **(A,B)** Patient 5, The tumor squeezed bilateral cavernous sinuses (yellow arrow); the proportion of the suprasellar part volume of the tumor is about 60%. The suprasellar tumor contained a cyst (Yellow triangle) and compressed the third ventricle (red arrow). **(C,D)** Three months after the operation, the residual tumor was located above the sellar area and partially sunk into the sellar area (red arrow).

The parameters with *P* < 0.05 in univariate analysis were included in the binary multivariate logistic analysis. It was found that a higher Knosp grade [OR = 0.214, 95% confidence interval (CI): 0.05–0.917; *P* = 0.038], greater proportion of tumor suprasellar volume (OR = 0.937, 95% CI: 0.898–0.978; *P* = 0.003), and intraoperative evaluation of GPA invading the cavernous sinus (OR = 0.187, 95% CI: 0.039–0.898; *P* = 0.036) were the significant risk factors that make it more difficult to achieve GTR. Therefore, these three parameters can be used as independent risk factors to evaluate whether a GPA can be completely removed with microscopic TSS. The results are presented in Table 4. Figure 2 shows that CSI limits total tumor resection, and Figure 3 shows that a greater proportion of tumor suprasellar volume limits total tumor resection.

**Table 4:** Binary logistic regression analysis of the risk factors affecting the degree of GPA resection.

**Risk factors**	**Odds Ratio**	**95% CI**	***P*-value**
Intraoperative confirmed CSI	0.187	0.039, 0.898	0.036^*^
Knosp grade	0.214	0.05, 0.917	0.038^*^
Proportion of suprasellar tumor volume	0.937	0.898, 0.978	0.003^*^
Invasion of the middle cranial fossa	0.197	0.020, 1.913	0.161

*CI, confidence interval; CSI, cavernous sinus invasion; GPA, giant pituitary adenomas. Significant P-values are shown in bold*.

* *P < 0.05*.

## Discussion

GPA is a rare, slow-growing, and histologically benign tumor. Visual impairment and hydrocephalus are often the first symptoms of GPA (9). Generally, microsurgery or endoscopic surgery *via* the nasal transsphenoidal approach can effectively and safely remove giant adenomas with smooth structures without massive intracranial expansion or CSI (10).

Cappabianca et al. proposed that the size of the tumor is not important, and the intracranial growth pattern of the tumor is the most important factor when evaluating the possibility of endoscopic surgery for large tumors and GPA (11). The results of the present study confirm this view. There was no significant difference in the tumor diameter and suprasellar tumor height between the two groups. However, the larger the proportion of the GPA in the suprasellar part, the smaller the degree of possible resection. This may be because the index of “suprasellar volume proportion of the tumor” can better reflect the mode of growth of the tumor. The higher the degree of suprasellar expansion, the farther away from the tubular field of vision under the microscope, which becomes the blind area in the operator's field of vision. When the operator cannot guarantee that the operation can fully protect the optic nerve, choroidal artery, and other structures outside the field of vision or under insufficient lighting, the part of the tumor on the sella is often left. However, in this study, the suprasellar height of the tumor seemed unrelated to the degree of resection. Perhaps because the “suprasellar height” cannot accurately reflect the shape of the suprasellar tumors, the “thick” tumors in the suprasellar part may be more difficult to sink than the “thin” tumors. The compressed sellar diaphragm gradually decreases during tumor resection, and residual tumors often form in the folds. It may be difficult for the operator to find the residual tumor in the sellar diaphragm fold under a microscope. Postoperative MRI often shows a residual tumor attached to the sellar diaphragm. In addition, the compliance of the suprasellar tumors and intraoperative intracranial pressure also affect the smooth sinking.

Koutourousiou et al. reported that the real limitation of the transnasal transsphenoidal approach is that the pituitary adenoma has a multilobular structure and breaks through the medial wall of the cavernous sinus, resulting in CSI (12). In the present study, Knosp grade 3–4 and intraoperative confirmed CSI were the independent risk factors limiting the degree of GPA resection. These two indicators reflect the degree of lateral invasion of the GPA in the sella. Pituitary adenomas of Knosp grade 3–4 are generally regarded as invading the cavernous sinus. Fang et al. evaluated the reliability of Knosp grade (kg) and modified it to predict CSI and found that the rates of Knosp grade 2 and grade 3A tumors were close at 30 and 44%, respectively, which were significantly lower than those of Knosp grade 3 tumors (81%). However, the sensitivity of Knosp grade 3–4 in predicting CSI was only 61% (13). This may be why only 37 among the 53 patients with Knosp grade 3–4 GPA in this study were found to have CSI by intraoperative exploration. Univariate analysis showed that both the Knosp grade and intraoperative invasion of the cavernous sinus could predict the degree of GPA resection. It is generally believed that the integrity of the medial wall of the cavernous sinus during intraoperative exploration is the gold standard for evaluating the invasion of the cavernous sinus (14). Although there are different ways to evaluate intracavernous invasion, the GTR rate of GPA with intracavernous invasion is lower. In addition, although endoscopy improves the visualization of the surgical area, many previous reports have proposed that invasion of the cavernous sinus remains the most important factor limiting the GTR of the tumors using endoscopic TSS (15). In our recent surgical approach for Knosp 4 grade GPA, we tried to expand the sellar base to both sides and remove large tumors in the cavernous sinus through the anterior wall of the cavernous sinus under the microscope, resulting in satisfactory GTR. Residual tumors in the cavernous sinus restrict the therapeutic effect of surgery and reduce the postoperative biochemical remission rate of functional pituitary adenomas. This continuously damages health and greatly increases the economic and psychological burden on patients. Therefore, surgeons aim to improve the degree of resection of the tumors in the cavernous sinus during TSS.

The diameter, blood supply, texture, degree of boundary adhesion, and extension of the anterior and posterior sellar regions did not affect the degree of GPA resection. Furthermore, a study on the endoscopic resection of GPA through TSS reported that the anterior cranial fossa, posterior cranial fossa, or tumor size did not negatively impact the degree of resection (16). It is generally believed that CSF leakage occurs during the operation because the thin and deformed sellar diaphragm is pulled during the operation, resulting in damage or tear of the sellar diaphragm and leakage of CSF from the intracranial subarachnoid space above the sellar region into the sellar region. GPA often protrudes above the sellar region to varying degrees and expands on the sellar region, causing compression of the lateral and third ventricles and even hydrocephalus. Therefore, since the preoperative intracranial pressure is high, the high intracranial pressure can cause the tumor in the suprasellar part to sink slowly into the sellar area during TSS, enabling its removal. We believe that the high intracranial pressure is reduced by the early intraoperative CSF leakage, which affects the subsidence of large tumors on the sella. This results in a large number of residues in the suprasellar region. In particular, leakage of a large amount of CSF during surgery will significantly improve the difficulty of intraoperative resection. This study did not show that intraoperative CSF leakage impacted the resection degree of GPA, which may be related to the small intraoperative CSF leakage in this series of cases. The effect of a small amount of CSF leakage on the release of intracranial pressure may not be significant. The surgeon can remove the suprasellar tumor before the CSF is released in large quantities. Moreover, by increasing the end-tidal carbon dioxide tension and positive end-expiratory pressure, the intracranial pressure is increased, so as to force the tumor in the suprasellar part to sink.

There are many complications associated with GPA, including hypothalamic injury, pituitary dysfunction, diabetes insipidus, visual deterioration, oculomotor nerve paralysis, cerebrospinal fluid rhinorrhea, meningitis, and cerebral infarction (3). The most serious postoperative complication is residual tumor stroke, associated with a high mortality rate (17). A meta-analysis reported that postoperative residual tumor bleeding complications had decreased to 1.45% (18); however, it can still lead to disastrous consequences, such as intracranial nerve paralysis, intracranial infection, and disturbance of consciousness. Therefore, total or maximum resection of the tumor should be achieved as far as possible to ensure safety. If GTR cannot be achieved, bleeding from the residual tumor may occur within 1–2 days after surgery. We advocate that patients with GPA should be kept in the ICU for monitoring and treatment after surgery with close observation of the vital signs and routine cranial computed tomography examination within 1 day after surgery. In most cases, the sellar floor opening for microscopic TSS is small. At the end of the operation, there is no need to use the nasal mucosal valve to reconstruct the sellar floor. Only gelatin sponge and biological tissue protein glue can be used to block the sellar floor. We believe that this is an optimal method for reducing nasal complications.

Resection of GPA through transsphenoidal or transcranial surgery or combined surgery to the greatest degree possible aims to reduce the compression of the visual pathway and reduce the tumor volume as much as possible to obtain maximum control of the tumor (19). No differences in the tumor resection rate and postoperative biochemical remission rate between endoscopic and microscopic TSS were found in multiple meta-analyses (20, 21), even though the incidence of vascular complications in the endoscopic group was higher than that in the microscopic group (22). Elshazly et al. reported that the near GTR rate of GPA by endoscopic endonasal approach (resection degree ≥ 90%) was 47% (16). In the present study of GPA resection by microscopic TSS, the degree of GPA resection was ≥90% in 36 cases (49%). Therefore, there may be no significant difference in the degree of GPA resection between the microscopic TSS and endoscopic endonasal approach. Considering the minimally invasive nature of TSS, most patients choose this operation for treatment. However, this method has certain limitations. For example, a tumor with a dumbbell shape, irregular extension, or wrapping of the intracranial artery limits the safe or satisfactory resection of the tumor (23). Transsphenoidal and transcranial approaches should be selected flexibly according to the characteristics of the tumor. In some cases, combined surgery can maximize tumor resection and reduce the risk of swelling and bleeding from the residual tumor (5). Twenty patients with GPA were excluded from this study and underwent craniotomy, of which six patients underwent GTR, and 14 (70%) underwent subtotal resection. Consequently, to improve the resection rate, reduce complications, and improve the prognosis, we must amass more experience with transsphenoidal and multiple transcranial techniques (2).

## Conclusion

A higher proportion of suprasellar volume of GPA, invasion of the cavernous sinus confirmed during surgery, and high Knosp Steiner grade are the independent risk factors affecting the degree of resection of GPA by TSS under a microscope. Preoperative MRI showed that the tumor volume in the suprasellar region accounted for a high proportion of the GPA volume. TSS combined with craniotomy can improve the GTR rate of GPA. Choosing a single or combined surgical method for different GPAs may help improve the GTR rate.

## Data Availability Statement

The original contributions presented in the study are included in the article/supplementary material, further inquiries can be directed to the corresponding authors.

## Ethics Statement

The studies involving human participants were reviewed and approved by the Ethics Committee at Fujian Medical University. The patients/participants provided their written informed consent to participate in this study.

## Author Contributions

ZJP, JW, SM, TF, and SW conceived and designed the study and wrote the manuscript. LW, YF, SY, MW, and JW analyzed the data and prepared tables. All authors contributed to the article and approved the submitted version.

## Funding

The present study was funded by the Fujian Provincial Key Project of Science and Technology Plan (Grant No. 2019Y9045), The Fujian Medical University Sailing Fund Project (Grant No. 2020QH2040), and The Fujian Medical University Sailing Fund Project (Grant No. 2020QH2043).

## Conflict of Interest

The authors declare that the research was conducted in the absence of any commercial or financial relationships that could be construed as a potential conflict of interest.

## Publisher's Note

All claims expressed in this article are solely those of the authors and do not necessarily represent those of their affiliated organizations, or those of the publisher, the editors and the reviewers. Any product that may be evaluated in this article, or claim that may be made by its manufacturer, is not guaranteed or endorsed by the publisher.

## Abbreviations

CSF: cerebrospinal fluid
CSI: cavernous sinus invasion
GPA: giant pituitary adenomas
GTR: gross-total resection
MRI: magnetic resonance imaging
TSS: transsphenoidal surgery.
